# Aortic arch cannulation with the guidance of transesophageal echocardiography for Stanford type A aortic dissection

**DOI:** 10.1186/s13019-018-0779-5

**Published:** 2018-10-11

**Authors:** Hao Ma, Zhenghua Xiao, Jun Shi, Lulu Liu, Chaoyi Qin, Yingqiang Guo

**Affiliations:** Department of Cardiovascular Surgery, West China Hospital, Sichuan University, Chengdu, 610041 China

**Keywords:** Cannulation site, Aortic arch cannulation, Transesophageal echocardiography, Femoral cannulation, Stanford type a aortic dissection

## Abstract

**Background:**

Aortic arch cannulation for an antegrade central perfusion during the surgery for Stanford type A aortic dissection can be performed within median sternotomy. We summarize the safety and convenient profile of the central cannulation strategy using the guidance of transesophageal echocardiography (TEE) in comparison to traditional femoral cannulation strategy.

**Methods:**

Sixty-two patients with acute Stanford type A aortic dissection underwent aortic arch surgery in our hospital. All the patients were operated by the same surgeon. Cannulation was performed in 33 patients through the aortic arch under the guidance of TEE (Group A) and in 29 patients through the femoral artery (Group F). Under moderate hypothermic circulatory arrest, the brain is continuously perfused in an anterograde manner through the brachiocephalic and left common carotid arteries. Preoperative characeristics and surgical information were collected for each patient. Additionally, 30-day mortality rate and the incidence of the temporary neurological dysfunction were recorded as the outcomes. To compare the categorical variables, we used the chi-squared test. Continuous variables were compared using the t-test.

**Results:**

Preoperative characteristics were almost similar between the two groups. The mean operation time (7.33 ± 1.14 h vs. 8.93 ± 2.59 h, *P* = 0.002) and the mean cardiopulmonary bypass (CPB) time (260.97 ± 45.14 min vs. 298.28 ± 95.89 min, *P* = 0.024) were significantly shorter in Group A than those in Group F. The 30-day mortality rates were 9.09 and 27.59% in Groups A and F, respectively (*P* = 0.057). And the incidences of temporary neurological dysfunction were 39.39 and 65.52% in Group A and F, respectively (*P* = 0.040).

**Conclusions:**

Aortic arch cannulation with the guidance of TEE during the aortic arch surgery is a simple, fast, safe, and less invasive technique for establishing cardiopulmonary bypass for Stanford type A aortic dissection.

## Background

Stanford type A aortic dissection is a devastating event associated with major morbidity and mortality and requires immediate surgical repair. During surgery for type A aortic dissection, the choice of cannulation site is of great importance to improve the outcomes of the operation [1]. For the past decades, various cannulation sites have been used. Femoral arterial cannulation (FC) has been used for cardiopulmonary bypass since the 1950s [2] and it has been reported to be the standard cannulation site, but it can bring a risk of distal re-entry, perfusion of the false lumen, malperfusion syndrome and cerebral embolization because of retrograde perfusion in the dissected aorta [3, 4]. Axillary arterial cannulation was firstly described by Villard et al. in 1976, but it was infrequently used for arterial inflow until 1995 when the Cleveland Clinic published positive results in 35 patients after axillary arterial cannulation [5]. It provides antegrade cerebral perfusion to reduce the risk of stroke and retrograde embolization. However, it also involves some local complications such as injury of the artery or the brachial plexus which can lead to arm ischemia, insufficient CPB flow, atherosclerosis of the artery, and often requires side graft sewn to the vessel [1, 6]. Subclavian artery cannulation is a more time consuming procedure and provides a cumbersome antegrade cerebral perfusion (ACP) because of selective ACP through only the right carotid artery during periods of systemic circulatory arrest [7]. What’s more, if the type A aortic dissection extends beyond the brachiocephalic artery, or if the patient has an incomplete circle of Willis, the surgeons would choose not to cannulate via sites like subclavian artery, innominate artery or axillary artery [4, 8]. Transapical aortic cannulation is an old technique that was initially described in the early 1970s [9], but it is limited to those patients with severely calcified ascending aortas and easy to bleed at the access site [10]. Recently, direct cannulation into the dissected ascending aorta has been reported by several surgeons [11–13] and that it can be performed rapidly without an additional incision. During the early 20th century, several surgeons tried to combine transesophageal echocardiography (TEE) with arterial cannulation to reduce the risk of cannulating into the false lumen [14, 15]. These techniques have been described in numerous studies and have been widely used. However, the question on which cannulation site is the optimal site remains controversial.

The present study was undertaken to compare the experience and results in patients undergoing surgery for Stanford type A aortic dissection using two different cannulation sites: the aortic arch under the guidance of TEE and the femoral artery. We compare the two methods and try to provide helpful information regarding the selection of the cannulation method for aortic arch surgery.

### Patient selection for surgery

This retrospective study was approved by the Institutional Review Board. Individual patient consent was not required. All patients with Stanford type A aortic dissection irrespective of the dissection flap in our hospital underwent computed tomography angiography (CTA) and transthoracic echocardiography (TTE) for diagnosis and operative planning. Cannulation sites were decided individually upon patient status and surgeon preference. From December 2015 to April 2017, 62 patients with acute Stanford type A aortic dissection underwent aortic arch surgery in our hospital. All the patients were operated by the same surgeon. Cannulation was performed in 33 patients through the aortic arch with the guidance of TEE (Group A) and in 29 patients through the femoral artery (Group F). Almost all of the 33 patients in Group A were complicated cases, wherein other conventional cannulation methods were precluded because of the involvement of the axillary and femoral arteries by the dissection flap. Clinical backgrounds and preoperative clinical condition of the patients are presented in Table 1.

**Table 1:** Preoperative patient characteristics

Variable	Group A(*n* = 33)	Group F(*n* = 29)	Total(*n* = 62)	*P* value
Age(means±S.D.)	46.48 ± 10.32	47.90 ± 9.93	47.15 ± 10.08	0.756
Male	29(87.88%)	21(72.41%)	50(83.33%)	0.124
BMI	26.05 ± 4.25	23.32 ± 3.11	24.77 ± 3.97	0.060
Smoke	23(69.70%)	11(37.93%)	54.84(%)	0.012
Drink	18(54.55%)	6(20.69%)	38.71(%)	0.006
Marfan’s syndrome	2(6.06%)	3(10.34%)	5(8.06%)	0.658
Hypertension	19(57.58%)	11(37.93%)	30(48.39%)	0.122
Coronary heart disease	2(6.06%)	1(3.45%)	3(4.84%)	1.000
Respiratory disease	2(6.06%)	2(6.90%)	4(6.45%)	1.000
Liver dysfunction	6(18.18%)	4(13.79%)	10(16.13%)	0.902
Renal dysfunction	4(12.12%)	3(10.34%)	7(4.84%)	1.000
Chronic aortic dissection	5(15.15%)	5(17.24%)	10(16.13%)	1.000
History of aortic dissection	2(6.06%)	0	2(3.23%)	0.494
Cardiac reoperation	2(6.06%)	0	2(3.23%)	0.494

### Surgical technique

After general anesthesia and intubation, standard median sternotomy was performed, and cardiopulmonary bypass (CPB) was instituted by cannulating either the aortic arch with the guidance of TEE (Group A) or the femoral artery (Group F).

#### Aortic arch cannulation technique

**Fig. 1:**
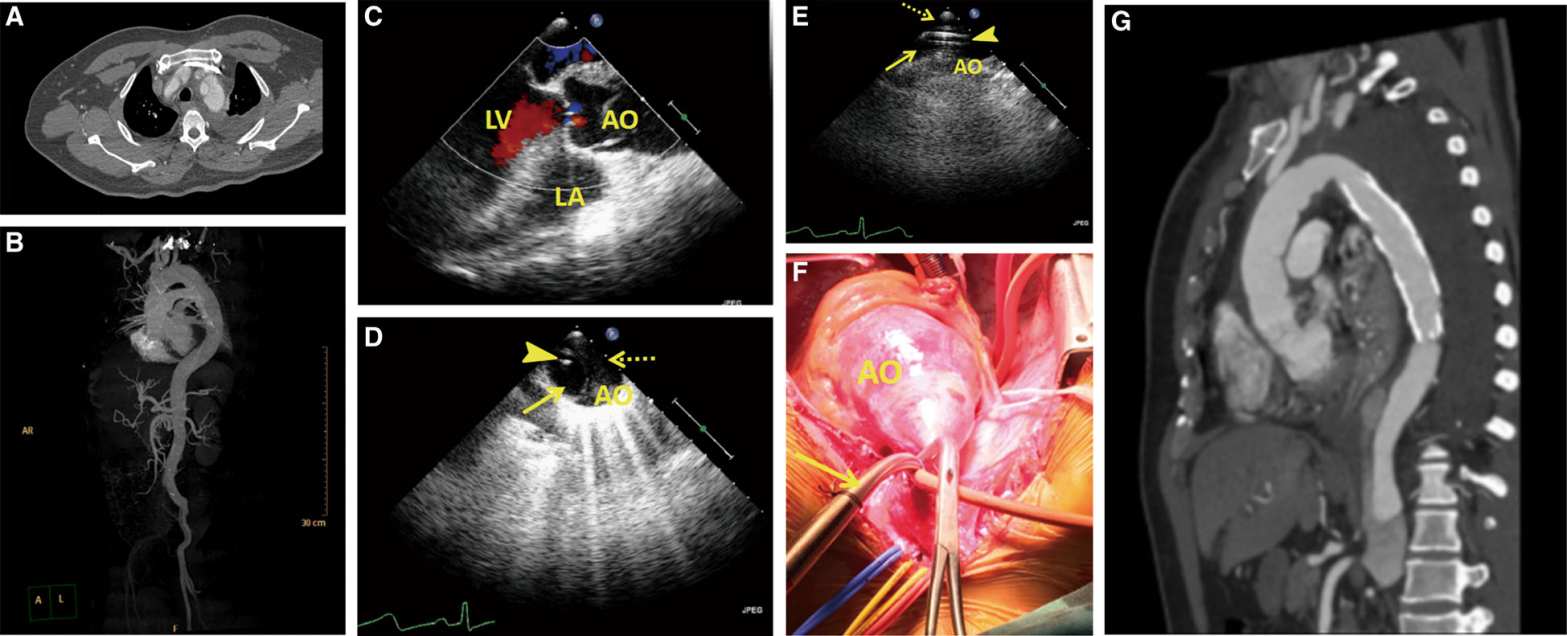
Perioperative images. **a**-**b** Computed tomography angiography (CTA) before the operation revealing a Stanford type A aortic dissection extending from the aortic root to the bilateral iliac artery. **c** Transesophageal echocardiography (TEE) showing a mild aortic regurgitation, an enlarged root (47) and ascending aorta (48–52), and an ejection fraction of 69%. **d** Transesophageal echocardiography (TEE) image. TEE showing the guide wire (arrow-head) present in the true lumen (arrow) of the descending aorta. The false lumen is depicted by a dotted-arrow. **e** Transesophageal echocardiography (TEE) image. TEE confirming the accurate positioning of the cannulation into the true lumen (arrow). Arrow-head is the false lumen. **f** The 20 Fr cannulation (Medtronic, arrow) advanced over the guide wire and cannulated into the true lumen of the aortic arch. **g** Postoperative CTA image revealing the patency of the three-branched vessels and the optimal position of the graft. Perioperative image. Computed tomography (CTA) before the operation revealing a Stanford type A aortic dissection extending from the aortic root to the bilateral iliac artery. Transthoracic echocardiography (TEE) image. Transthoracic echocardiography (TEE) showing a mild aortic regurgitation, an enlarged root (47) and ascending aorta (48–52), and an ejection fraction of 69%. Transesophageal echocardiography (TEE) image. TEE showing the guide wire (arrow-head) present in the true lumen(arrow) of the descending aorta. The false lumen is depicted by a dotted-arrow. Transthoracic echocardiography (TEE). TEE confirming the accurate positioning of the cannulation into the true lumen (arrow). Arrow-head is the false lumen. Transthoracic echocardiography (TEE). The 20 Fr cannulation (Medtronic, arrow) advanced over the guide wire and cannulated into the true lumen of the aortic arch. Postoperative CTA image. Postoperative CTA image revealing the patency of the three-branched vessels and the optimal position of the graft

Group A received aortic arch cannulation with the guidance of TEE, where in a TEE probe was inserted through the esophagus. Following the median sternotomy and systemic heparinization, the pericardium was opened slowly. A concentric pledget reinforced purse-string suture was placed through the adventitial layer on the lesser curvature of the aortic arch. A modified Potts Courmand style 18 gauge needle was used to puncture the aorta inside the purse-string suture. Once pulsatile bleeding was confirmed, a 0.035-in. flexible guide wire was introduced through the needle. After TEE confirmed the presence of the guide wire in the true lumen of the descending aorta, the needle was taken out, and the cannula was advanced over the guide wire. TEE confirmed the accurate positioning of the cannulation into the true lumen. After double-stage cannulas were inserted into the superior and inferior venae cavae, a CPB was established, and the patient started to cool down. The TEE was performed by cardiologists (Fig. 1)

#### Femoral cannulation technique

Group F received femoral cannulation. Cannulation of the right or the left femoral artery was surgically exposed prior to sternotomy. The venous cannulation was performed with a double-stage cannula via the right atrium. Then, a CPB was established, the patient started to cool down, and a standard median sternotomy was performed.

We used selective antegrade cerebral perfusion whenever total arch replacement was required, and the brain was selectively antegrade perfused with a rate of 5 ml/kg/min and a temperature of 25 °C to 27 °C. An ice pack was applied on the head to maintain cerebral hypothermia until CPB was restarted. Myocardial protection was obtained by means of an antegrade infusion of cold blood cardioplegia. The patients underwent David and Bentall operations, ascending aortic replacements, total aortic arch replacements, hemi-arch replacements, descending aortic stented elephant trunk implantation, or other operations (Table 2).

**Table 2:** Techniques used

Technique	Group A(n = 33)	Group F(n = 29)	Total(n = 62)	*P* value
Total arch replacement	31(93.94%)	20(68.97%)	51(82.26%	0.010
Hemiarch repair	0	6(20.69%)	6(9.68%)	0.008
Aortic debranching	2(6.06%)	1(3.45%)	3(4.83%)	1.000
Ascending aorta replacement	33(100%)	29(100%)	62(100%)	–
Elephant trunk	33(100%)	19(65.52%)	52(83.87%)	0.001
Aortic valve replacement	15(45.45%)	13(44.83%)	28(45.16%)	0.961
Aortic valve plastic	15(45.45%)	7(24.14%)	22(35.48%)	0.080
Coronary artery bypass	2(6.06%)	1(3.45%)	3(4.83%)	1.000
Mitral valve plastic	3(9.09%)	1(3.45%)	4(6.45%)	0.616
Tricuspid valve plastic	2(6.06%)	3(10.34%)	5(8.06%)	0.658
Repair of ruptured sinus of Valsalva aneurysm	0	1(3.45%)	1(1.61%)	0.468
Left vertebral artery reconstruction	0	1(3.45%)	1(1.61%)	0.468
Repair of auricular septal defect	1(3.03%)	0	1(1.61%)	1.000

CPB time was defined as the cumulative time on full-body CPB, including moderate hypothermic circulatory arrest (MHCA). MHCA time was defined as the cumulative time of full-body circulatory arrest, which is equivalent to the brain perfusion time. Operation time was defined as the time from incision to closure. Cross time was defined as the time from clamping the aorta to opening the aorta. Stroke was defined as a new postoperative focal neurologic deficit or cerebral hemorrhage that persisted for more than 72 h, or a new focal lesion of the brain detected by a computed tomography scan. Temporary neurologic dysfunction was defined as a focal neurologic deficit lasting for less than 72 h, or postoperative delirium, agitation, confusion, or decreased level of consciousness without any new structural abnormality observed on imaging [16, 17].

### Statistical analysis

Patient data were analyzed using SPSS 22.0 for Windows. Categorical variables are presented as numbers and percentages, and continuous variables are presented as mean and standard deviation values. To compare the categorical variables, we used the chi-squared test. Continuous variables were compared using the t-test.

## Methods

Sixty-two patients with acute Stanford type A aortic dissection underwent aortic arch surgery in our hospital. All the patients were operated by the same surgeon. Cannulation was performed in 33 patients through the aortic arch under the guidance of TEE (Group A) and in 29 patients through the femoral artery (Group F). Under moderate hypothermic circulatory arrest, the brain is continuously perfused in an anterograde manner through the brachiocephalic and left common carotid arteries. Preoperative characeristics and surgical information were collected for each patient. Additionally, 30-day mortality rate and the incidence of the temporary neurological dysfunction were recorded as the outcomes. To compare the categorical variables, we used the chi-squared test. Continuous variables were compared using the t-test The Methods include the sentences mentioned above and the part of the surgery process in the text.

## Results

### Patient characteristics

A total of 62 patients were diagnosed with acute Stanford type A aortic dissection by contrast-enhanced computer tomography and echocardiography, and they underwent elective ascending aortic surgery from December 2015 to April 2017. Patient characteristics are presented in Table 1. No significant differences in age, gender, body mass index (BMI), Marfan’s syndrome, hypertension, coronary heart disease, respiratory disease, liver dysfunction, renal dysfunction and cardiac reoperation were found. However, the rates of patients who smoke (69.70% vs. 37.94%, *P* = 0.012) and drink (54.55% vs. 20.69%, *P* = 0.006) were higher in Group A than in Group F. Liver dysfunction was defined as the assay index of laboratory examination that was used to evaluate the liver function were unusual. Renal dysfunction was defined as the value of creatinine > 120 mmol/L.

### Intraoperative parameters

We determined that the puncture and cannulation of the aortic arch were possible in 33 of the 62 patients, and none of them experienced intraoperative difficulties. Additionally, femoral cannulation was performed in the remaining 29 patients, except that two patients required another cannulation through the innominate artery because of the presence of malperfusion in their right arms when the ascending aortas were cross-clamped. The techniques used are shown in Table 2. All the patients underwent ascending aorta replacement. Hemiarch repair was performed in 6 patients (0% vs. 20.69%, *P* = 0.008), whereas total arch replacement was performed in 51 patients (93.04% vs. 28.97%, *P* = 0.010). Aortic debranching was performed in 3 patients (6.06% vs. 3.45%, *P* = 1.000). A total of 52 patients underwent elephant trunk procedure (100% vs. 5.52%, *P* = 0.001); 28 patients needed aortic valve replacement because of severe aortic valve regurgitation (45.45% vs. 44.83%, *P* = 0.961); and 22 patients needed aortic valve plastic surgery (45.45% vs. 24.14%, *P* = 0.080). Concomitant cardiac procedures included coronary artery bypass in 4.84% (*n* = 3) of the patients because the coronary arteries were affected by the dissection, mitral valve plastic surgery in 6.45% (*n* = 4), tricuspid valve plastic surgery in 8.06% (*n* = 5), repair of ruptured sinus of Valsalva aneurysm in 1.61% (*n* = 1), left vertebral artery reconstruction in 1.61% (*n* = 1), and repair of atrial septal defect in 1.61% (*n* = 1). In general, we concluded that the surgeries were more complicated in Group A.

Surgical duration and intraoperative data summary are shown in Table 3. After starting the extracorporeal circulation, the body temperature was cooled to 25 °C to 27 °C in all patients (nasopharyngeal temperature of 25.49 °C ± 2.07 °C vs. 26.05 °C ± 2.78 °C, *P* = 0.259; anal temperature of 27.14 °C ± 1.73 °C vs. 27.36 °C ± 2.64 °C, *P* = 0.144). No significant differences in MHCA time, absence of circulatory arrest, Hct after CPB, minimum hemoglobin concentration, and maximum serum lactic acid concentration during operations were found. However, the mean operation time (7.33 ± 1.14 h vs. 8.93 ± 2.59 h, *P* = 0.002) and the mean CPB time (260.97 ± 45.14 min vs. 298.28 ± 95.89 min, *P* = 0.024) were significantly shorter in Group A than in Group F. One patient required a second run of extracorporeal circulation to stop the hemorrhage after the termination of extracorporeal circulation.

**Table 3:** Intraoperative variables

Variables	Group A(n = 33)	Group F(n = 29)	Total(n = 62)	*P* value
Operation time(h)	7.33 ± 1.14	8.93 ± 2.59	8.08 ± 2.10	0.002
CPB time(min)	260.97 ± 45.14	298.28 ± 95.89	278.42 ± 75.11	0.024
Cross time(min)	170.67 ± 41.72	193.55 ± 57.97	181.37 ± 50.87	0.089
The lowest temperature during CPB(°C)				
Nasopharyngeal temperature	25.49 ± 2.07	26.05 ± 2.78	25.75 ± 2.42	0.259
Anal temperature	27.14 ± 1.73	27.36 ± 2.64	27.24 ± 2.18	0.114
MHCA time(min)	40.97 ± 7.98	37.00 ± 9.39	39.28 ± 8.75	0.287
Absence of circulatory arrest	2(6.06%)	6(20.69%)	8(12.90%)	0.131
Hct after CPB (%)	27.92 ± 4.14	26.62 ± 4.95	27.31 ± 4.55	0.463
Maximum internal time of twice myocardial perfusion during CPB(min)	71.70 ± 14.80	69.71 ± 24.69	70.77 ± 19.90	0.001
Minimun hemoglobin concentration during operation(g/L)	77.23 ± 15.48	74.35 ± 10.18	75.88 ± 13.24	0.849
Maximum serum lactic acid concentration during operation(mol/L)	9.06 ± 4.70	10.34 ± 6.27	9.66 ± 5.48	0.192

*CPB* cardiopulmonary bypass, *MHCA* moderate hypothermic circulatory arrest

### Postoperative parameters

Table 4 shows the postoperative parameters. The length of intensive care unit (ICU) stay (5.50 ± 3.35 vs. 4.62 ± 1.75, *P* = 0.200) and intubation time (43.54 ± 36.38 vs. 36.52 ± 27.54, *P* = 0.393) were similar in both groups as the same with the need for tracheostomy (9.09% vs. 6.90%, *p* = 1.000), thoracentesis (30.30% vs. 44.83%, *p* = 0.237), and thoracic cavity closed-chest drainage (6.06% vs. 3.45%, *p* = 1.000). No significant intergroup differences existed in the frequency of hemorrhage requiring rethoracotomy, which occurred in only one patient in Group A; sepsis, which occurred in one patient (3.03%) in Group A and in one patient (3.45%) in Group F; renal failure, which occurred in two patients (6.06%) in Group A and in three patients (10.34%) in Group F; multiple organ failure, which occurred in two patients (6.06%) in Group A and in two patients (6.90%) in Group F; circulatory failure, which occurred in one patient (3.03%) in Group A and in four patients (13.79%) in Group F; intestinal ischemia, which occurred in one patient (3.45%) in Group F; limb ischemia, which occurred in one patient (3.45%) in Group F; or rehospitalization, which occurred in one patient (3.45%) in Group F only. The rate of temporary neurological dysfunction (TND) was significantly lower in Group A than in Group F (39.39% vs. 65.52%, *p* = 0.040) and the wake time was significantly shorter in Group A than in Group F (7.22 ± 3.78 vs. 12.35 ± 12.64, *p* = 0.046). No statistical difference in in-hospital mortality was found between the two groups; however, a trend toward a lower 30 day mortality (9.09% vs. 27.59%, *p* = 0.057) was observed in Group A.

**Table 4:** Postoperative variables

Variables	Group A(n = 33)	Group F(n = 29)	Total(n = 62)	*P* value
Length of ICU stay(days)	5.50 ± 3.35	4.62 ± 1.75	5.12 ± 2.79	0.200
Fail to come out of ICU	5(15.15%)	8(27.59%)	13(20.97%)	0.230
Re-enter ICU	3(9.09%)	1(3.45%)	4(6.54%)	0.616
Wake time(h)	7.22 ± 3.78	12.35 ± 12.64	9.59 ± 9.29	0.046
Fail to wake	5(15.15%)	5(17.24%)	10(16.13%)	1.000
Intubation time(hours)	43.54 ± 36.38	36.52 ± 27.54	40.37 ± 32.57	0.393
Fail to remove the intubation	5(15.15%)	6(20.69%)	11(17.74%)	0.569
Tracheostomy	3(9.09%)	2(6.90%)	5(8.06%)	1.000
Length of remove chest tube(days)	11.48 ± 3.90	9.30 ± 3.70	10.52 ± 3.93	0.913
Chest tube drainage(ml/24 h)	688.63 ± 363.03	715.31 ± 435.82	701.31 ± 396.12	0.385
Fail to remove chest tube	4(12.12%)	6(20.69%)	10(16.13%)	0.569
Thoracentesis	10(30.30%)	13(44.83%)	23(37.10%)	0.237
Thoracic cavity closed drainage	2(6.06%)	1(3.45%)	3(4.84%)	1.000
Hemorrhage requiring rethoracotomy	1(3.03%)	0	1(1.61%)	1.000
Sepsis	1(3.03%)	1(3.45%)	2(3.23%)	1.000
Temporary neurological dysfunction	13(39.39%)	19(65.52%)	32(51.61%)	0.040
Stroke	3(9.09%)	1(3.45%)	4(6.54%)	0.616
Renal failure	2(6.06%)	3(10.34%)	5(8.06%)	0.658
Circulatory failure	1(3.03%)	4(13.79%)	5(8.06%)	0.176
Multiple organ failure	2(6.06%)	2(6.90%)	4(6.54%)	1.000
Intestinal ischemic	0	1(3.45%)	1(1.61%)	0.468
Limb ischemic	0	1(3.45%)	1(1.61%)	0.468
30-day-mortality	3(9.09%)	8(27.59%)	11(17.74%)	0.057
Rehospitalization	0	1(3.45%)	1(1.61%)	0.468

## Discussion

The optimal cannulation site for the repair of acute Stanford type A aortic dissection remain unknown. The most common site for cannulation in this setting was the femoral artery until the late 1990s [18]. However, femoral artery cannulation has a risk of distal re-entry, false lumen perfusion, organ malperfusion, and cerebral embolization because of retrograde perfusion in the dissected aorta [3, 4]. As an alternative cannulation technique, direct ascending cannulation has been advocated by the Hannover group [19] and has been developed through the guidance of TEE by some surgeons [20, 21]. Our center considered FC as the normal cannulation technique in the repair of the Stanford type A aortic dissection and has begun to use aortic arch cannulation with the guidance of TEE since 2015.

Aortic arch cannulation with the guidance of TEE is easy, fast, and straightforward, and ensures antegrade flow in the aorta and could be advantageous compared with the axillary and femoral cannulations. If the three branches of the aortic arch and bilateral femoral arteries are all affected by the aortic dissection, this proves to be the best procedure to cannulate through the aortic arch. Opening another surgical area is not required, thus establishing CPB becomes faster, which is highly beneficial to a patient experiencing hemodynamic instability. Moreover, no additional incisions are required and surgeons do not need to repair the cannulation site, thereby avoiding injuries in other peripheral arteries. With the guidance of TEE, we did not introduce perfusion of the false lumen because the cannulation was directly inserted into the false lumen. Surgoens can use a large-diameter cannulation to provide sufficient perfusion during CPB and shorten the time of cooling the body temperature, cutting down the time of surgery as a whole. What’s more, this cannulation technique tends to provide selective antegrade cerebral perfusion to protect the brain from edema, stroke and other neurological complications and retrograde cerebral embolization. However, this technique has one negative outcome, which is the risk of aortic rupture at the cannulation site. Khaladj et al. [19] reported that only 1 of 122 patients (0.8%) had an aortic rupture caused by aortic cannulation in patients with Stanford type A aortic dissection. Hiroyuki et al. [18] did not report aortic ruptures after aortic cannulation of 82 patients for 20 years. Moreover, we did not cause any aortic rupture at the cannulation site in the 33 patients in the study. Therefore, the danger of aortic rupture at the cannulation site is extremely low.

In Group A, the aortic arch cannulation with the guidance of TEE was technically feasible and safe in all 33 patients. Using careful and safe cannulation techniques, we encountered no difficulties related to the cannulation procedure and did not transfer to a different cannulation site. We did not observe any malperfusion phenomenon or problems directly related to aortic arch cannulation. However, in Group F, we found intraoperative malperfusion of the right upper limb, as evidenced by the decreased blood pressure of the right radial artery; and of the left brain hemisphere, as evidenced by the decreased cerebral oxygen saturation. This phenomenon may be caused by the malperfusion of the innominate artery. We suspected that this phenomenon may have been caused by the following: (1) the diameter of the cannula, which was limited by the diameter of the femoral artery, was too small to provide enough blood for the upper limbs and the brain; and (2) some blood may have flowed into the false lumen after the CPB was started; thus, the perfusion flow was lower than the value that detected by the instrument. This phenomenon required no treatment other than the additional cannulation inserted into the innominate artery. Moreover, intestinal ischemia was detected through the abdominal computed tomography (CT) scan of one of the two patients. The patients were fasted for more than 1 week and were given parenteral nutrition. Furthermore, no femoral arterial rupture was present in these patients. A patient’s left dorsalis pedis artery pulsation was non-palpable during the first week post-operation and muscle force was weaker in the left lower limb than in the right lower limb. The temperature was lower in the left lower limb than in the other parts of the body. These results may have been caused by malperfusion because the cannulation was inserted into the left femoral artery of this patient.

Mortality with acute Stanford type A aortic dissection remains high with an average 30 day mortality rate of approximately 17%, which progressively increases to 25% in octogenarians [22]. In our single-center study, we reported a 30 day mortality rate of 17.74% in 62 patients. Our study shows that aortic arch cannulation with the guidance of TEE has a positive effect on 30 day mortality. Stefan and colleagues [23] found that the cannulation strategy used for the initial bypass has no impact on mortality, even though the femoral cannulation is performed more often in a sick patient group, as categorized by ASA classification. In another study, the risk for early mortality was driven by the preoperative clinical and hemodynamic status before the operation rather than by the cannulation technique [24]. In the retrospective study of Masahiro and colleagues [1], the mean operative time, mean CPB time, and interval time between the start of operation and start of CPB was significantly shorter in the central group, and central cannulation had a positive effect on mortality (6.8% vs. 17.3%, *p* < 0.001). In conclusion, their study showed that a direct central cannulation through the ascending aorta is successful in repairing type A dissection and produced surgical results that are superior to those of femoral cannulation. Hiroyuki and colleagues [18] found a trend toward a reduced mortality rate in patients with aortic cannulation, although no statistical differences in postoperative mortalities and morbidities between the aortic and femoral cannulation groups were present. The large German Registry for acute aortic dissection (GERAADA) [25] with 2137 patients does not show significant influence of the cannulation site on any outcome parameter.

Preoperative and postoperative neurologic symptoms were present in approximately 7 and 20%, respectively, of the patients [22]. In our study, preoperative neurologic symptoms did not differ between the two groups, but a significantly lower rate of TND was present in Group A than in Group F. Moreover, the patients who received aortic arch cannulation with the guidance of TEE tended to recover quicker than those who received the femoral cannulation. This effect will prevent cerebral embolization because of retrograde perfusion in the dissected aorta caused by the femoral cannulation. The risk of stroke between the two groups did not differ. With adequate cerebral perfusion and cerebral monitoring using the bilateral cerebral oxygen, a moderate hypothermic arrest with temperatures between 25 °C and 27 °C is acceptable in both groups. In the study by Stefan and colleagues [23], no differences in neurologic symptoms regarding the perfusion strategy were found. In another singer-center study by Stefan and colleagues [24], their data showed a new neurologic event in 11% of all patients, which did not differ between femoral and central cannulation. In other studies [1, 18, 24], the rate of short-term and postoperative neurology in patients receiving different cannulation techniques did not differ.

Moreover, the mean operation time and mean CPB time were significantly shorter in Group A than in Group F. This result may be attributed to the aortic arch cannulation with the guidance of TEE, which does not require another incision; and to the flow of the cannulation, which is larger than that of the femoral cannulation. Moreover, LV asynergy or pseudoaneurysm on the apex and aortic valve regurgitation in the early postoperative period by TTE were absent.

## Conclusion

Direct aortic arch cannulation using the Seldinger technique with the guidance of TEE may be a simple, accurate, fast, and safe cannulation technique to establish CPB during the surgery to treat type A aortic dissection. This technique is an appropriate approach for patients with peripheral arteries affected by the aortic dissection or those with hemodynamic instability.

### Limitation

Limitations of the present study are the relatively small number of patients from a single institution and the non-randomized and retrospective study design. Moreover, the cannulation site was not randomly chosen but individually decided depending on patient status. Therefore, further studies with large patient populations are necessary.

## Abbreviations

ACP: Antegrade cerebral perfusion
BMI: Body mass index
CPB: Cardiopulmonary bypass
CT: Computed tomography
CTA: Computed tomography angiography
FC: Femoral arterial cannulation
GERAADA: German Registry for acute aortic dissection
ICU: Intensive care unit
MHCA: Moderate hypothermic circulatory arrest
TEE: Transesophageal echocardiography
TND: Temporary neurological dysfunction
TTE: Transthoracic echocardiography
